# Prevention and Treatment of Skin Pigmentation Disorders

**DOI:** 10.3390/jcm13154312

**Published:** 2024-07-24

**Authors:** Ziad Khamaysi, Badea Jiryis

**Affiliations:** 1 Laser Unit, Department of Dermatology, Rambam Health Care Campus, Haifa 3109601, Israel; 2 Bruce Rappaport Faculty of Medicine, Technion Institute of Technology, Haifa 3525433, Israel

Pigmentation disorders are conditions that affect the color of a person’s skin. These disorders can manifest as areas of skin that are hyperpigmented or hypopigmented, or in some cases, patches of skin that have lost their pigment entirely. This Editorial aims to provide a brief overview of current treatments for pigmentation disorders, starting with vitiligo as the main hypopigmentation disorder, and then continuing with hyperpigmentation disorders.

Vitiligo is a chronic condition characterized by the loss of skin pigmentation, affecting approximately 0.5–2% of the global population. This condition can greatly impact the quality of life for those affected. Recent years have seen significant advancements in our comprehension of how vitiligo develops. It is now understood to be influenced by a complex interplay of genetics, oxidative stress, inflammation, and environmental factors. Standard treatments for vitiligo encompass camouflage, topical corticosteroids, topical calcineurin inhibitors, oral corticosteroids, phototherapy, and surgical interventions. The choice of treatment depends on the patient’s preferences and specific characteristics. With a deeper understanding of the role of the Janus kinase (JAK)/signal transducer and activator of transcription (STAT) pathway in vitiligo’s development, treatment options have expanded to include the first cream approved by the US FDA for repigmenting vitiligo patients [1].

Several small studies and individual case reports have shown positive results when using JAK inhibitors to treat vitiligo, whether applied topically or taken orally, often alongside phototherapy. On the other hand, there is limited and inconclusive information in the literature regarding the effectiveness of biologics in treating vitiligo, with only a handful of cases reporting successful outcomes.

Moving to the hyperpigmentation group of disorders, we chose to start with melasma, a common dermatological condition characterized by the presence of hyperpigmented patches and macules, that typically manifests on the facial skin [2]. The treatment of melasma continues to pose a challenge due to the limited clinical effectiveness, high recurrence rates, and the occurrence of adverse reactions associated with many of the treatments. The management of melasma typically commences with preventive measures involving sun protection followed by a range of treatments that encompass topical applications, oral therapies, chemical peels, microneedling, laser procedures, and light treatments [3,4].

A Review paper from this Special Issue provided a comprehensive and detailed overview of the treatment landscape for dermal pigmentation in skin of color patients; the study covered a spectrum of conditions and provided insights into difficulties and hopeful treatment methods. Its discussion emphasized the interactions between lasers and tissues, agreed-upon suggestions, and the potential for treatment in cases where traditional methods are not effective. Various laser technologies and their uses have been discussed, emphasizing the continual search for the best protocols, safety measures, and effectiveness in dermatological laser therapies. The insights gained from the authors’ experience in a cohort of 122 Indian patients further validate the use of sequential laser therapy in treating dermal pigmentation in SoC, pointing towards future research and clinical applications [5].

There is limited information about the age-dependent changes in the skin color of body parts other than the face. Lee E et al. analyzed the differences in skin color between various body parts and the changes in skin color of each body part with age. The observations revealed that the skin tones of the elbows and knees appeared darker compared to other areas of the body, and that the skin tone of the forearms and upper arms tended to darken with age. As a result, they recommend focusing on skincare for the elbows, knees, forearms, and upper arms to address the darkening caused by the natural movements and exposure to external factors like sunlight, UV rays, pollution, and friction. While redness decreased with age in most body regions except the forearms and upper arms, further research is necessary to determine whether this change is linked to reduced blood circulation or other physiological or pathological alterations [6].

In the management of post-inflammatory hyperpigmentation, it is crucial to address underlying inflammatory skin conditions and practice sun-protective behaviors. When it comes to treatment options, the standard guidelines for treating post-inflammatory hyperpigmentation involve topical agents like hydroquinone, retinoids, steroids, azelaic acid, and vitamin C, either alone or in combination. In cases where patients do not respond well to topical treatments or could benefit from additional therapy, procedures such as chemical peels and lasers can be considered. However, there is a risk of worsening hyperpigmentation, particularly in darker skin tones, with some of these treatments. Therefore, efforts should be made to minimize adverse effects by using suitable topical formulations and concentrations, along with superficial chemical peels containing salicylic acid or low-energy Q-switched Nd:YAG laser therapy as required [7].

There are many treatment options for hyperpigmentation, including topical, systemic or procedural options. The initial approach in treating hyperpigmentation typically involves the use of topical lightening agents. The application of energy-based devices is generally reserved for patients with refractory or recurrent lesions or those seeking swift resolution of their condition. However, using these devices as monotherapy can sometimes worsen hyperpigmentation and result in rebound lesions after discontinuation of treatment. As a result, combining energy-based devices with topical lightening agents is recommended. This combination approach offers a higher response rate, a shorter duration of treatment, lower side effects (such as post-inflammatory hyperpigmentation), and a reduced recurrence rate. It is advisable to maintain topical therapy along with an increased number of sessions with energy-based devices to preserve treatment efficacy and minimize the likelihood of recurrence.

In the future, treating skin hyperpigmentation with lasers is expected to become even more precise and effective. Advanced laser technologies will continue to evolve, providing tailored treatments for different types and depths of pigmentation. The use of machine learning and artificial intelligence may further enhance treatment outcomes by analyzing skin characteristics and optimizing laser settings. Additionally, non-ablative laser procedures with minimal downtime are likely to become more popular, allowing for convenient and quick treatment sessions. Overall, the future of treating skin hyperpigmentation with lasers appears promising, offering patients safer, more personalized, and efficient solutions.
